# Social Networks of Adolescents and Young Adults with Cancer: A Cross-Sectional Study

**DOI:** 10.3390/curroncol32090502

**Published:** 2025-09-09

**Authors:** Rohini R. Datta, Bojana Petrovic, Argerie Tsimicalis, A. Fuchsia Howard, Emily K. Drake, Sheila N. Garland, Karine Chalifour, Norma M. D’Agostino, Abha A. Gupta, Jacqueline L. Bender

**Affiliations:** 1 Dalla Lana School of Public Health, University of Toronto, Toronto, ON M5T 3M7, Canada; rohini.datta@mail.utoronto.ca (R.R.D.); bojana.petrovic@alumni.utoronto.ca (B.P.); 2 Department of Supportive Care, Princess Margaret Cancer Center, University Health Network, Toronto, ON M5G 2C4, Canadaabha.gupta@uhn.ca (A.A.G.); 3 Sinai Health, Toronto, ON M5T 3L9, Canada; 4 Ingram School of Nursing, McGill University, Montreal, QC H3A 2M7, Canada; argerie.tsimicalis@mcgill.ca; 5 School of Nursing, University of British Columbia, Vancouver, BC V6T 2B5, Canada; fuchsia.howard@ubc.ca; 6 Interdisciplinary Health Studies Program, Faculty of Science, Mount Allison University, Sackville, NB E4L 1E4, Canada; edrake@mta.ca; 7 Department of Psychology, Faculty of Science, Memorial University, St. John’s, NL A1C 3X9, Canada; sheila.garland@mun.ca; 8 Discipline of Oncology, Faculty of Medicine, Memorial University, St. John’s, NL A1B 3X5, Canada; 9 Young Adult Cancer Canada, St. John’s, NL A1B 3K3, Canada; karine@youngadultcancer.ca; 10 Division of Medical Oncology and Hematology, Princess Margaret Cancer Centre, University Health Network, Toronto, ON M5G 2C4, Canada

**Keywords:** adolescents and young adults, social networks, social network index, digital social network, cancer

## Abstract

A cancer diagnosis disrupts the social networks of adolescents and young adults (AYAs), impacting their overall health and wellbeing. This cross-sectional study examined the social network integration (SNI; size and frequency of contact) of AYAs with cancer in Canada. A survey was distributed to AYAs with cancer at an urban cancer centre and across Canada (n = 334). SNI was measured with the Berkman–Syme Social Network Index (SNI) and a modified version accounting for online interactions (SNI+). A multivariable logistic regression analysis was performed to identify factors associated with SNI and SNI+. A total of 54.8% and 68% of AYAs with cancer were classified as socially integrated with each measure, respectively. Living with others was associated with greater SNI and SNI+ (SNI OR = 3.27, 95% CI = 1.39, 7.72; SNI+ OR = 2.52, 95% CI = 1.14, 5.58), and an annual personal income of >CAD 80,000 was associated with greater SNI+ (SNI+ OR = 2.92, 95% CI = 1.09, 7.77). A significant proportion of AYAs with cancer are socially isolated. AYAs with cancer who live alone and whose personal income is less than CAD 80,000 are at a higher risk of social isolation. Digital technology could be leveraged to increase the SNI of AYAs with cancer.

## Simple Summary

A cancer diagnosis can affect the social networks of adolescents and young adults (AYAs) with cancer, which in turn impacts their health and wellbeing. This study looked at how connected AYAs with cancer are to their social networks—the number of people they interact with and how often. A total of 334 AYAs with cancer completed a survey that examined their social network integration using two measures, the standard Berkman–Syme Social Network Index (SNI) and a modified version that included online interaction (SNI+). The study found that about 55% of AYAs with cancer were socially integrated with the standard measure, but this rose to 68% when online interactions were included. AYAs who lived with others and whose personal income was greater than CAD 80,000 were more socially integrated. The results suggest that many AYAs with cancer are socially isolated, especially those who live alone or who have lower incomes. Using digital technology could help increase the social network integration of AYAs with cancer.

## 1. Introduction

An estimated 9000 adolescents and young adults (AYAs) aged 15 to 39 were diagnosed with cancer in 2023, representing about 4% of the cancer population [1]. A cancer diagnosis during adolescence and young adulthood can be detrimental to key life events such as the establishment of independence, the formation of identity, the development of relationships, the completion of education, and the attainment of careers [2]. AYAs diagnosed with cancer struggle to achieve these milestones while coping with the significant physical, emotional, and social impacts of cancer and its treatment, resulting in psychosocial needs that are distinct from other age groups [3,4]. The Canadian Task Force on Adolescents and Young Adults with Cancer concluded that the unique psychosocial needs of AYAs with cancer must be met for them to reach their full potential [5,6], making psychosocial care a priority in AYA oncology.

Social networks have a powerful impact on health and wellbeing [7] and could be leveraged to address some of the psychosocial needs of AYAs with cancer. Social networks are the web of social relationships that surround an individual and the characteristics of the ties within those relationships [8]. A cancer diagnosis can significantly disrupt the social networks of AYAs as the diagnosis often leads to feelings of social isolation, loneliness, difficulty maintaining relationships, and a need for additional support that existing social relationships may not be able to provide [9]. An individual’s social network can provide different levels of access to resources, referrals, advice, and opportunities that can influence cancer outcomes by impacting the timeliness of diagnosis and treatment as well as treatment and self-management decisions, which can impact cancer mortality [10]. Social networks can also influence health behaviours both positively and negatively, and these behaviours can spread across social networks [11,12,13]. Furthermore, chronic stress resulting from social isolation can influence cancer progression through increased levels of inflammatory mediators [14].

Most research on the social networks of patients with cancer and cancer survivors has focused on the social networks of paediatric and adult cancer survivors [15,16,17,18,19,20]. Among adult cancer survivors, significant associations have been found between social networks and outcomes such as quality of life and mortality [16,17,19,21,22]. Social network integration (SNI), which is the degree to which an individual is connected to a broad range of social relationships [8], is associated with better quality of life among long-term survivors of leukemia, lymphoma, and colorectal cancer, with social support being an important mediator [19,21]. SNI has also been associated with improved prognosis and survival among women diagnosed with breast cancer [16,17,22].

There are only two known studies of the social networks of AYA cancer survivors, both of which were conducted in the United States. One study found that AYA cancer survivors had significantly larger functional social network indexes (FSNI) compared to age- and sex-matched controls [23]. In addition, survivors of lymphoma, leukemia, and solid tumours had larger FSNI scores compared to survivors of central nervous system (CNS) malignancies. Factors associated with a larger FSNI score included planning for the future, the use of emotional and instrumental support, positive reframing, religious engagement, less behavioural disengagement, and less denial [23]. Another study compared the FSNI scores of AYA cancer survivors with age- and sex-matched controls [24]. Low FSNI scores were indirectly associated with poor physical functioning, anxiety, and depression through poor social support and loneliness [24].

To our knowledge, no prior studies have accounted for online interactions when assessing SNI. The Internet and social media are a critical source of social interaction used by young people in general [25] and those with cancer to stay connected with peers, obtain health information, and find support [26]. However, social network measures used to date have either not specified the interaction medium or focused on in-person or telephone-mediated interactions with network members. Given the critical role of the Internet and social media in an AYA’s cancer experience, we undertook an analysis of the social networks of Canadian AYAs with cancer that accounted for online social interactions. For this, we modified the commonly used Berkman–Syme Social Network Index [27] to account for online interactions. Additionally, to build on the limited research on the social networks of AYAs, we sought to identify the factors associated with SNI among AYAs with cancer in Canada. The findings of this study can be used to identify AYAs at risk of experiencing the negative effects of poor SNI and thus inform AYA-specific psychosocial oncology care.

## 2. Materials and Methods

### 2.1. Study Design and Setting

The Connect 4 Health study was a mixed-method study of the peer support and navigation needs of AYAs diagnosed with cancer [28]. It involved a cross-sectional survey that was administered in-person to AYAs diagnosed with cancer at the Princess Margaret Cancer Center in Toronto, Ontario, Canada, and online to reach AYAs with cancer across Canada between September 2018 and April 2019. The survey results are reported following the Strengthening the Reporting of Observational Studies in Epidemiology (STROBE) guidelines [29]. Detailed recruitment methods can be found elsewhere [28]. Ethics approval was obtained from the University Health Network Research Ethics Board (UHN REB # 18-5386).

### 2.2. Participants

Eligible patients were those who had received a cancer diagnosis between the ages of 15 to 39, were able to read and speak English, and were actively receiving treatment or were within 10 years of receiving cancer treatment. For this study, only survey respondents who indicated that they lived in Canada and responded to all questions pertaining to social networks were included in the analysis.

### 2.3. Variables and Measures

#### 2.3.1. Questionnaire

The questionnaire consisted of 57 questions that examined the following four topics: (A) Cancer-Specific Background Information, (B) Preferences for Peer Support from Other AYAs Diagnosed with Cancer, (C) Overall Health and Wellbeing, and (D) Background Information. The questionnaire is available in Supplementary File S1. This analysis used data obtained from Sections A, C, and D. Findings from Section B were reported in a prior publication [28].

#### 2.3.2. Social Network Index

Social networks were evaluated using the original and a modified version of the Berkman–Syme Social Network Index, which was developed to summarise the relationship between social contacts and mortality [27]. The Berkman–Syme Social Network Index assesses social integration based on 4 domains: (1) relationship status, (2) the number and frequency of interaction with close contacts (e.g., friends and relatives) (i) seen or (ii) talked to by telephone at least once per month; (3) belonging to a social group or organisation; and (4) frequency of attendance at religious or spiritual meetings. The question regarding marital status was modified to include the following options: single, married or living with a life partner, or in a relationship but not living with a life partner. SNI was computed using the original Berkman–Syme Index and a modified version (SNI+) that accounted for online interactions with the addition of two questions to the close contacts domain: How many close friends do you communicate with by text, email or other ways online at least once a month? and How many close relatives do you communicate with by text, email or other ways online at least once a month?. For both outcomes, responses in each of the four domains were dichotomised and summed, resulting in a total SNI score for each measure, in following with previous studies [30,31,32,33]. The scoring instructions for the SNI measures can be found in Supplementary File S2. Both SNI and SNI+ outcomes had a categorical score ranging from 0 to 4, with 0 or 1 representing social isolation, 2 representing moderate social isolation, 3 representing moderate social integration, and 4 representing social integration. For the purposes of this analysis, the outcomes were dichotomised with a score of 0 to 1 representing social isolation and a score of 2, 3, or 4 representing social integration, as performed in previous research [34,35].

#### 2.3.3. Sociodemographic and Clinical Variables

Potential covariates were determined a priori. They included sociodemographic factors, clinical factors, and psychosocial factors. Sociodemographic factors included age, education, employment status, household living arrangements, number of dependent children, sex, gender, sexual orientation, race and ethnicity, and annual personal income. Clinical factors included age at diagnosis, time since diagnosis, cancer type, and treatment type. Psychosocial factors included anxiety, measured with the Generalised Anxiety Disorder 7-Item (GAD-7) [36], depression measured with the Patient Health Questionnaire-9 (PHQ-9) [37], loneliness measured with the UCLA Loneliness Scale [38], social support measured with the Social Provisions Scale [39], and coping self-efficacy measured with the Cancer Behaviour Inventory [40].

For the analysis, age was treated as a continuous variable. Education, sexual orientation, annual personal income, and race and ethnicity were collapsed into binary categories due to low cell counts. Highest level of education was dichotomised as “secondary school or less” and “more than secondary school”. Sexual orientation was dichotomised as “heterosexual” and “homosexual, bisexual, and fluid”. Annual personal income was dichotomised as “less than $80,000 (CAD)” and “$80,000 (CAD) or more”. For race and ethnicity, the category of “White” included participants who self-selected White/European/North American. The category of “racial and ethnic minority” included participants who self-selected African, Arab, Black, Caribbean, Chinese, Filipino, Indigenous, Japanese, Latin American, South Asian, Southeast Asian, West Asian, or “other”.

### 2.4. Statistical Analysis

Analysis was performed using SPSS version 28 [41]. Descriptive statistics were calculated, measures of central tendencies were computed for continuous variables, and proportions were computed for categorical variables.

For both outcomes, analysis of the missing data was conducted to ensure there were no statistically significant differences between the participants who responded and those who did not. Chi-square analysis was conducted for the categorical variables and *t*-tests for the continuous variables, with *p* < 0.05 indicating a significant association. Cells were either collapsed or removed from the analysis to address low variability, defined as less than 5% cell counts.

Univariable logistic regression analysis for both outcomes was performed for all potential covariates. Covariates found to be significantly associated with either or both outcomes were included in the final multivariable logistic regression models. Associations of *p* < 0.05 were considered significant. Variables that had more than 10% of their data missing were excluded from the multivariable analysis. The variables included in the final models were assessed for multicollinearity as well as the presence of influential points. Finally, the proposed models were validated using a bootstrap validation method.

## 3. Results

### 3.1. Participant Characteristics

Of the 434 participants who responded to the questionnaire, 334 individuals (76.9%) completed the questions required for this analysis (Table 1). Respondents were on average 30.8 years of age (SD = 6.2 years), and most identified as women (64.4%), heterosexual (85.6%), and white (61.1%). Most respondents also had completed post-secondary education (71.6%), were unemployed (80.2%), were living in an urban/suburban location (86.5%), were living with others (87.7%), and had no dependent children (74.3%). Nearly half (48%) reported a personal income of less than CAD 40,000. The average time since diagnosis was 39.3 months (SD = 46.0), the most common cancer type was breast cancer (17.4%), and the most common treatment type was drug/chemotherapy (72.2%). Most respondents were categorised as having minimal anxiety (45.8%), based on their GAD-7 score, and minimal depression (49.7%), based on their PHQ-9 score.

### 3.2. SNI

Over 50% of respondents were classified as socially integrated with each SNI measure (e.g., scored 2, 3, or 4) (Table 2). However, more respondents were classified as socially integrated with the SNI+ measure (68%, n = 219) in comparison to the SNI measure that did not account for online interactions (54.8%, n = 177), reflecting a 23.7% increase in the number of socially integrated AYAs with cancer.

### 3.3. Social Network Index Domains

Nearly two-thirds (61.1%) of respondents were either married, living with a life partner, or in a relationship. Slightly more than one-third (34.7%) had 13 or more close contacts per month, which included family members and close friends contacted in-person and by telephone. When online interactions were included as a method of contact, there was an 82.8% increase in the number of respondents with 13 or more close contacts per month. Over half (53.3%) of respondents indicated some form of group/community participation, and 62.6% indicated they never participated in religious activities (Table 3).

### 3.4. Univariable Analysis

Univariable logistic regression was performed to assess the association with all potential covariates and SNI and SNI+, respectively (Table 4). Several sociodemographic variables were associated with both SNI and SNI+. These included having an education greater than secondary school (SNI OR = 1.78, 95% CI = 1.09, 2.91; SNI+ OR = 2.15, 95% CI = 1.29, 3.56), living in a home with others (SNI OR = 3.26, 95% CI = 1.59, 6.68; SNI+ OR = 3.03, 95% CI = 1.55, 5.95), having dependent children (SNI OR = 2.48, 95% CI = 1.46, 4.22; SNI+ OR = 2.50, 95% CI = 1.37, 4.58), and having a personal income of CAD 80,000 or more (SNI OR = 3.19, 95% CI = 1.59, 6.42; SNI+ OR = 4.20, 95% CI = 1.72, 10.23). Age was only associated with SNI (SNI OR = 1.04, 95% CI = 1.01, 1.08), whereas sexual orientation was only associated with SNI+ (SNI+ OR = 0.45, 95% CI = 0.22, 0.93).

Of the psychosocial variables analysed, having minimal depression (score of 0–4 on the PHQ-9) was associated with social integration (SNI *p*-value = 0.005; SNI+ *p*-value = 0.032). Higher levels of coping behaviour were also associated with being socially integrated (SNI OR = 1.02, 95% CI = 1.01, 1.03; SNI+ OR = 1.01, 95% CI = 1.00, 1.02) but had over 10% of the data missing and therefore was not considered for the multivariable analysis. None of the clinical variables were associated with social integration.

### 3.5. Multivariable Analysis

A multivariable logistic regression model was built for SNI and SNI+ respectively, using the covariates identified to be significant in the unadjusted model as well as gender and race/ethnicity given their conceptual relationships with social networks (Table 5) [39,40,41,42]. In the adjusted model, living in a household with others was associated with both outcomes (SNI OR = 3.27, 95% CI = 1.39, 7.72; SNI+ OR = 2.52, 95% CI = 1.14 5.58). In addition, having an annual personal income of CAD 80,000 or greater was significantly associated with social integration in the model that accounted for online interactions (SNI+ OR = 2.92, 95% CI = 1.09, 7.77). However, there was a lower magnitude of association between social integration and living arrangements in the model that accounted for online interactions.

## 4. Discussion

### 4.1. Key Findings

This study expands our understanding of the social networks of AYAs diagnosed with cancer and is the first known study to account for online interactions in the measurement of SNI. Greater SNI was observed when online interactions were included as a method of contact, suggesting that technology has the potential to increase the extent to which AYAs diagnosed with cancer are socially integrated. Several factors were individually associated with both social integration measures, including higher education, living with others, having children under the age of 18, an annual personal income of CAD 80,000 or more, and greater coping self-efficacy. The factors that remained significant in the adjusted models were living with others and an annual personal income of CAD 80,000 or more when online interactions were included in the social integration measure. These findings suggest that AYAs diagnosed with cancer who live alone or who have lower income are at higher risk of social isolation and should be provided with psychosocial support that leverages online resources to promote SNI.

### 4.2. Interpretation of Findings

#### 4.2.1. High Proportion of AYAs with Cancer Who Are Socially Isolated

Nearly half (45%) of the AYAs with cancer in this sample would be considered socially isolated with the original Berkman–Syme SNI measure, and one-third (32%) would be considered socially isolated with the modified SNI measure that included online interactions. In comparison, a 2025 U.S. study that used the Berkman–Syme measure to assess social integration in a large sample of primary care patients (n = 73,373) ranging in age from 18 to 99 reported that on average, 28.69% of the residents were socially isolated [42]. Of note, social integration scores covaried strongly and symmetrically with health-related variables. Patients with a health condition were more likely to be socially isolated, and being socially isolated also increased the odds of having a health condition.

#### 4.2.2. Technology Could Increase Social Integration Among AYAs with Cancer

The greater proportion of AYAs with cancer who were socially integrated when online interactions were considered reflects the significant role that technology and social media plays in the lives of AYAs [43]. Through social media, AYAs can access support from other young adults with cancer as well as from their offline support network [43]. The convenience and accessibility of social media removes the need to commute to interact with peers, which can be challenging for AYAs who feel unwell or who depend on their parents for transportation, while providing anonymity if desired. Online interactions may also increase the frequency of in-person interactions. A Canadian study found that weekly online interactions with friends and family were associated with more frequent in-person interactions [25]. A prior study of patients diagnosed with cancer at the same hospital found that social media provides access to relevant information and educational resources and can serve as a helpful distraction from cancer [44]. However, a qualitative study revealed that some AYAs may be uncomfortable disclosing their cancer diagnosis on social media, transitioning to media consumption, or withdrawing from social media use [45]. In addition, several studies have documented an association between problematic social media use and psychological distress in AYAs [46]. Hence, AYAs may need guidance on how to use social media effectively to support better coping and adjustment with cancer.

#### 4.2.3. Factors Associated with Social Integration

Several sociodemographic factors were associated with both measures of social integration in the univariable analyses, including completing higher education, living with others, having children, and having higher personal income. However, only living with others and personal income were associated with social integration in the multivariable analyses. The 2025 U.S. study of social integration among primary care patients also found an association between higher socioeconomic status and higher social integration scores. Unlike our study, they also found disparities in the prevalence of social isolation based on race, ethnicity, sexual orientation, and sex [42]. Specifically, members of racialised groups, individuals identifying as gay or lesbian, and males experienced higher odds of social isolation. Further research is needed to explore the association between these sociodemographic factors and social isolation among patients living with cancer in Canada.

In our study, AYAs’ household living arrangements were associated with social integration as predicted, likely because of the impact of living with others on the number of interactions with close contacts per month and overall social connectedness [47]. Likewise, Kroenke reported that having children was positively associated with social integration among women with breast cancer [16,17]. Living with others has also been shown to enhance the quality of life of patients living with cancer [48] and may be critically important for the wellbeing of younger patients living with cancer. In their study, Rustoen et al. found that younger patients living with cancer (e.g., aged 19 to 39 years) who lived alone had a significantly lower quality of life compared to older patients who lived alone [48]. Disruption in social networks may be more challenging for younger patients who may have more fragile social networks and limited coping skills, negatively impacting their quality of life.

While personal income was associated with both measures of social integration in the univariable analyses, in the multivariable analysis, higher income was only associated with social integration when online interactions were considered. Cancer in young adulthood is a significant financial burden. In one study of Canadian AYAs living with cancer, 49% missed at least one year of work, and they were more likely to have outstanding debt and to not own assets compared to their non-cancer peers [49]. AYAs living with cancer are also more likely than AYAs without cancer to be unemployed [50]. Among patients living with cancer, the financial impact of a diagnosis and treatment has consequences on the social aspect of patients’ lives. Many patients experience short- and long-term impacts on their employment, which causes financial strain, leading to negative impacts on interpersonal relationships and social withdrawal [51]. The association between personal income and SNI when online interactions were included in the measure is likely because income influences the type and quality of Internet access (e.g., personal computer and highspeed broadband Internet) [52].

Unlike other studies examining the social integration of patients living with cancer, we did not find any association with clinical factors (e.g., cancer or treatment type). Among the adult breast cancer population, Kroenke et al. reported that social integration was associated with more intensive treatment (e.g., increased likelihood of chemotherapy and hormonal therapy as well as receiving radiation and tamoxifen with treatment) [53]. Likewise, Huang et al. and Poudel et al. found that among AYAs living with cancer, social integration was associated with being treated with chemotherapy [23,24]. Huang et al. also found differences in social integration based on cancer type among AYAs living with cancer. Specifically, having a CNS malignancy was associated with decreased social integration, which the authors hypothesised may be related to communication and social functioning challenges associated with neurocognitive impairment [23].

Lastly, of the psychosocial outcomes examined, only depression and coping skills were associated with social integration, but these did not remain significant when other variables were accounted for. Specifically, individuals without depression or minimal levels of depression and high coping behaviours were more likely to be socially integrated. These findings support the conclusions drawn by previous studies on the relationship between social integration and psychosocial functioning. Huang et al. reported that higher functional social integration was associated with better coping skills among AYAs with cancer [23]. Additionally, of all health-related factors examined, Ahmad et al. found that depression, which was associated with social isolation among primary care patients, explained most of the variance in social integration scores [42].

### 4.3. Strengths, Limitations, and Future Research

This study fills an important gap in the literature on the social networks of AYAs diagnosed with cancer. A major strength of this study was the creation of a modified social network index that includes online interactions with close contacts in one’s network. Based on the existing literature and scoring techniques of the BSNI, this modified social network index allows for an examination of the impact of online interactions on the social integration of AYAs diagnosed with cancer. Future research should test and validate this modified social network index in a larger sample. In addition, given that most psychosocial screening tools used in AYA cancer care to date do not assess social networks, further work is needed to develop clinically meaningful measures of social network integration to support screening efforts.

The cross-sectional design of the questionnaire is a limitation. As the temporality of the covariates and the outcome could not be determined from the data due to the study design, the factors included in the model were determined based on the existing literature. In addition, the study was limited to people who speak English. Further work is needed to translate and culturally adapt the social network index measure for use in other languages and cultures. Another limitation of this study is the extent of missing data for coping behaviours. Future research on this topic would also benefit from examining the network diversity of AYAs diagnosed with cancer and accounting for peer interactions with other cancer peers. Prior work has shown that feeling connected to the young adult cancer community can enhance post-traumatic growth among AYAs diagnosed with cancer [54].

## 5. Conclusions

Given the critical role of social relationships in health and wellbeing, screening for SNI could help to identify those at risk of social isolation. AYAs diagnosed with cancer who live alone and who have lower income should be considered at risk of social isolation and provided additional support. Online interactions can increase SNI among AYAs and could be leveraged to provide additional and accessible interventions to foster social connectedness.

## Figures and Tables

**Table 1:** Sociodemographic, clinical, and psychosocial characteristics of the full sample and by SNI measure.

Covariate	Full Sample (n = 334)	SNI (n = 323)	SNI+ (n = 322)
Age (Years)			
Mean (SD)	30.8 (6.2)	31.0 (6.1)	31.0 (6.1)
Highest Level of Education, n (%)			
Less than secondary school	8 (2.4)	7 (2.2)	7 (2.2)
Secondary school	87 (26.0)	83 (25.7)	83 (25.8)
Post-secondary degree	239 (71.6)	233 (72.1)	232 (72.0)
Household Living Arrangements, n (%)			
Live alone	41 (12.3)	40 (12.4)	40 (12.4)
Live with others	293 (87.7)	283 (87.6)	282 (87.6)
Dependent Children, n (%)			
Yes	86 (25.7)	85 (26.3)	85 (26.4)
No	248 (74.3)	238 (73.7)	237 (73.6)
Sex, n (%)			
Male	117 (35.0)	113 (35.0)	113 (35.1)
Female	215 (64.4)	208 (64.4)	207 (64.3)
Gender, n (%)			
Man	117 (35.0)	113 (35.0)	113 (35.1)
Woman	215 (64.4)	208 (64.4)	207 (64.3)
Sexual Orientation, n (%)			
Heterosexual	286 (85.6)	277 (85.8)	276 (85.7)
Homosexual	15 (4.5)	15 (4.6)	15 (4.7)
Bisexual	19 (5.7)	17 (5.3)	17 (5.3)
Fluid	1 (0.3)	1 (0.3)	1 (0.3)
Unsure	2 (0.6)	2 (0.6)	2 (0.6)
Other	2 (0.6)	2 (0.6)	2 (0.6)
Race and Ethnicity *, n (%)			
African	1 (0.3)	1 (0.3)	1 (0.3)
Arab	10 (3.0)	9 (2.8)	9 (2.8)
Black	1 (0.3)	1 (0.3)	1 (0.3)
Caribbean	5 (1.5)	5 (1.5)	5 (1.6)
Chinese	22 (6.6)	21 (6.5)	21 (6.5)
Filipino	12 (3.6)	11 (3.4)	11 (3.4)
Indigenous	4 (1.2)	4 (1.2)	4 (1.2)
Latin American	10 (3.0)	10 (3.1)	10 (3.1)
Multiracial	36 (10.8)	35 (10.8)	35 (10.9)
South Asian	18 (5.4)	18 (5.6)	18 (5.6)
Southeast Asian	2 (0.6)	2 (0.6)	2 (0.6)
West Asian	7 (2.1)	6 (1.9)	5 (1.6)
White	204 (61.1)	198 (61.3)	198 (10.9)
Other	1 (0.3)	1 (0.3)	1 (0.3)
Province or Territory, n (%)			
Newfoundland and Labrador	6 (1.8)	6 (1.9)	6 (1.9)
Nova Scotia	6 (1.8)	6 (1.9)	6 (1.9)
Quebec	11 (3.3)	11 (3.4)	11 (3.4)
Ontario	273 (81.7)	263 (81.4)	262 (81.4)
Manitoba	14 (4.2)	14 (4.3)	14 (4.3)
Saskatchewan	1 (0.3)	1 (0.3)	1 (0.3)
Alberta	13 (3.9)	12 (3.7)	12 (3.7)
British Columbia	9 (2.7)	9 (2.8)	9 (2.8)
Northwest Territories	1 (0.3)	1 (0.3)	1 (0.3)
Location Setting, n (%)			
Urban/Suburban	289 (86.5)	281 (87.0)	280 (87.0)
Town/Rural	44 (13.2)	42 (13.0)	42 (13.0)
Personal Income (CAD), n (%)			
No income	38 (11.4)	36 (11.1)	36 (11.2)
Less than CAD 20,000	68 (20.4)	66 (20.4)	66 (20.5)
CAD 2000 to less than CAD 40,000	54 (16.2)	53 (16.4)	53 (16.5)
CAD 4000 to less than CAD 60,000	52 (15.6)	49 (15.2)	49 (15.2)
CAD 6000 to less than CAD 80,000	34 (10.2)	34 (10.5)	34 (10.6)
CAD 80,000 or more	50 (15.0)	49 (15.2)	49 (15.2)
Time Since Diagnosis (Months)			
Mean (SD)	39.3 (46.0)	38.9 (45.4)	39.0 (45.4)
Cancer Type *, n (%)			
Breast	58 (17.4)	58 (18.0)	58 (18.0)
Gynaecological (cervical, uterine, ovarian)	23 (6.9)	23 (7.1)	23 (7.1)
Genitourinary (bladder, renal, testicular)	41 (12.3)	40 (12.4)	40 (12.4)
Hematologic (Hodgkin’s lymphoma, non-Hodgkin’s lymphoma, leukemia)	93 (27.8)	89 (27.6)	89 (27.6)
Gastrointestinal (colorectal, stomach, liver, pancreas, oesophageal)	24 (7.2)	21 (6.5)	20 (6.2)
Endocrine (thyroid, neuroendocrine, endocrine)	29 (8.7)	28 (8.7)	28 (8.7)
Sarcoma	31 (9.3)	30 (9.3)	30 (9.3)
Neurologic (brain, peripheral nervous system)	16 (4.8)	16 (5.0)	16 (5.0)
Skin (skin, melanoma)	10 (3.0)	10 (3.1)	10 (3.1)
Other	6 (1.8)	6 (1.8)	6 (1.8)
Treatment Type *, n (%)			
Drug or chemotherapy	241 (72.2)	232 (71.8)	231 (71.7)
Hormone therapy	52 (15.6)	52 (16.1)	52 (16.1)
Radiation therapy	137 (41.0)	130 (40.2)	129 (40.1)
Surgery	191 (57.2)	187 (57.9)	187 (58.1)
Bone marrow or stem cell transplant	19 (5.7)	17 (5.3)	17 (5.3)
Immunotherapy	12 (3.6)	12 (3.7)	12 (3.7)
Radioactive iodine	7 (2.1)	7 (2.2)	7 (2.2)
Other	8 (2.4)	8 (2.5)	8 (2.5)
None	8 (2.4)	8 (2.5)	8 (2.5)
Anxiety (GAD-7), n (%)			
Minimal (0–4)	153 (45.8)	148 (45.8)	148 (46.0)
Mild (5–9)	108 (32.3)	103 (31.9)	102 (31.7)
Moderate (10–14)	43 (12.9)	43 (13.3)	43 (13.4)
Severe (15–21)	26 (7.8)	25 (7.7)	25 (7.8)
Depression (PHQ-9), n (%)			
Minimal (0–4)	166 (49.7)	159 (49.2)	159 (49.4)
Mild (5–9)	85 (25.4)	83 (25.7)	83 (25.8)
Moderate (10–14)	43 (12.9)	43 (13.3)	42 (13.0)
Moderately Severe (15–19)	25 (7.5)	23 (7.1)	23 (7.1)
Severe (20–27)	9 (2.7)	9 (2.8)	9 (2.8)
Coping Self-Efficacy (Cancer Behaviour Inventory)			
Mean (SD)	135.8 (27.4)	135.1 (27.1)	135.2 (27.1)

Notes. CAD = Canadian dollars, SD = Standard deviation, SNI = Original Berkman–Syme Social Network Index measure, SNI+ = Modified Berkman–Syme Social Network Index measure accounting for online interactions. Denominator includes those who did not answer the question or indicated “prefer not to answer”. * Participants selected multiple responses if relevant. Percentages may not total 100 due to missing responses.

**Table 2:** Social network outcomes when accounting for different aspects of close contacts.

	Social Network Index	SNI (n = 323)	SNI+ (n = 322)
Isolated	Socially isolated (0–1)	146 (45.2)	103 (32.0)
Integrated	Moderately socially isolated (2)	108 (33.4)	125 (38.8)
Integrated	Moderately socially integrated (3)	60 (18.6)	76 (23.6)
Integrated	Socially integrated (4)	9 (2.8)	18 (5.6)

Notes. SNI = Original Berkman–Syme Social Network Index measure, SNI+ = Modified Berkman–Syme Social Network Index measure accounting for online interactions.

**Table 3:** Social network index domains of the full sample and by SNI measure.

Covariate	Full Sample (n = 334)	SNI (n = 323)	SNI+ (n = 322)
Marital Status, n (%)			
Single	129 (38.6)	126 (39.0)	126 (39.1)
Married or living with a partner	155 (46.4)	151 (46.7)	150 (46.6)
In a relationship, but not living with a life partner	49 (14.7)	46 (14.2)	46 (14.3)
Close Contacts ^a^, n (%)			
<13 Close contacts per month	214 (64.1)	211 (65.3)	-
≥13 Close contacts per month	116 (34.7)	112 (34.7)	-
Missing	4 (1.2)	0	-
Close Contacts ^b^, n (%)			
<13 Close contacts per month	117 (35.0)	-	116 (36.0)
≥13 Close contacts per month	212 (63.5)	-	206 (64.0)
Group Participation, n (%)			
No	150 (44.9)	146 (45.2)	146 (45.3)
Yes	178 (53.3)	177 (54.8)	176 (54.7)
Religious Participation, n (%)			
Never	209 (62.6)	201 (62.2)	201 (62.4)
≤every few months	74 (22.2)	72 (22.3)	71 (22.0)
≥once or twice a month	51 (15.3)	50 (15.5)	50 (15.5)

Notes. ^a^ In-person and telephone close contacts, ^b^ In-person, telephone, and online contacts. SNI = Original Berkman–Syme Social Network Index measure, SNI+ = Modified Berkman–Syme Social Network Index measure accounting for online interactions.

**Table 4:** Univariable logistic regression associated with SNI measures.

	SNI		SNI+	
Covariates	Odds Ratio (95% CI)	*p*-Value	Odds Ratio (95% CI)	*p*-Value
Age (Years)	1.04 (1.01, 1.08)	0.020	1.04 (1.00, 1.08)	0.067
Highest Level of Education				
Secondary school or less	Reference		Reference	
More than secondary school	1.78 (1.09, 2.91)	0.021	2.15 (1.29, 3.56)	0.003
Household Living Arrangements				
Live alone	Reference		Reference	
Live with others	3.26 (1.59, 6.68)	0.001	3.03 (1.55, 5.95)	0.001
Dependent Children				
No	Reference		Reference	
Yes	2.48 (1.46, 4.22)	<0.001	2.50 (1.37, 4.58)	0.003
Gender				
Man	Reference		Reference	
Women	1.00 (0.63, 1.58)	0.992	1.21 (0.74, 1.96)	0.455
Sexual Orientation				
Heterosexual	Reference		Reference	
Homosexual/Bisexual/Fluid	0.49 (0.23, 1.02)	0.058	0.45 (0.22, 0.93)	0.032
Race and Ethnicity				
White	Reference		Reference	
Racial and ethnic minoritised group	0.70 (0.45, 1.10)	0.120	0.72 (0.45, 1.16)	0.174
Personal Income (CAD)				
Less than CAD 80,000	Reference		Reference	
CAD 80,000 or more	3.19 (1.59, 6.42)	0.001	4.20 (1.72, 10.28)	0.002
Cancer Type		0.435		0.583
Breast	Reference		Reference	
Gynaecological	0.79 (0.30, 2.12)	0.645	0.65 (0.23, 1.85)	0.424
Genitourinary	0.37 (0.16, 0.84)	0.018	0.47 (0.20, 1.12)	0.087
Hematologic	0.82 (0.42, 1.61)	0.566	0.89 (0.42, 1.89)	0.767
Gastrointestinal	0.82 (0.30, 2.25)	0.692	0.65 (0.22, 1.93)	0.435
Endocrine	0.53 (0.21, 1.32)	0.172	0.87 (0.32, 2.39)	0.790
Sarcoma	0.70 (0.29, 1.70)	0.430	0.46 (0.18, 1.16)	0.098
Neurologic	0.79 (0.26, 2.41)	0.673	0.77 (0.23, 2.57)	0.668
Chemotherapy				
No	Reference		Reference	
Yes	1.25 (0.76, 2.03)	0.378	1.03 (0.61, 1.73)	0.925
Hormone Therapy				
No	Reference		Reference	
Yes	0.97 (0.53, 1.76)	0.915	0.79 (0.43, 1.48)	0.464
Radiation Therapy				
No	Reference		Reference	
Yes	0.96 (0.61, 1.50)	0.842	1.31 (0.80, 2.13)	0.271
Surgery				
No	Reference		Reference	
Yes	0.95 (0.61, 1.49)	0.830	1.01 (0.63, 1.63)	0.963
Bone Marrow Treatment				
No	Reference		Reference	
Yes	0.44 (0.16, 1.21)	0.110	0.51 (0.19, 1.37)	0.184
Anxiety		0.453		0.407
Minimal	Reference		Reference	
Mild	0.98 (0.59, 1.63)	0.944	1.31 (0.75, 2.28)	0.342
Moderate	0.66 (0.34, 1.31)	0.237	0.69 (0.34, 1.38)	0.291
Severe	0.60 (0.26, 1.41)	0.239	1.05 (0.42, 2.61)	0.913
Depression (PHQ-9)		0.005		0.032
Minimal (0–4)	Reference		Reference	
Mild (5–9)	0.61 (0.36, 1.04)	0.071	0.65 (0.37, 1.14)	0.130
Moderate (10–14)	1.11 (0.55, 2.22)	0.774	1.49 (0.66, 3.37)	0.334
Severe (15–27)	0.26 (0.11, 0.59)	0.001	0.41 (0.19, 0.88)	0.023
Coping Behaviour	1.02 (1.01, 1.03)	<0.001	1.01 (1.00, 1.02)	0.008

Notes. CAD = Canadian dollars, SNI = Original Berkman–Syme Social Network Index measure, SNI+ = Modified Berkman–Syme Social Network Index measure accounting for online interactions.

**Table 5:** Multivariable logistic regression for the factors associated with SNI measures.

	SNI		SNI+	
Covariates	Odds Ratio (95% CI)	*p*-Value	Odds Ratio (95% CI)	*p*-Value
Age	1.03 (0.97, 1.08)	0.364	1.00 (0.95, 1.05)	0.942
Highest Level of Education				
Secondary School or less	Reference		Reference	
More than Secondary School	1.20 (0.64, 2.25)	0.579	1.78 (0.94, 3.35)	0.076
Household Living Arrangements				
Live alone	Reference		Reference	
Live with others	3.27 (1.39, 7.72)	0.007	2.52 (1.14, 5.58)	0.023
Dependent Children				
No	Reference		Reference	
Yes	1.89 (0.96, 3.73)	0.066	1.71 (0.80, 3.63)	0.167
Gender				
Male	Reference		Reference	
Female	1.04 (0.58, 1.88)	0.885	1.20 (0.65, 2.22)	0.569
Sexual Orientation				
Heterosexual	Reference		Reference	
Homosexual/Bisexual/Fluid	0.65 (0.27, 1.57)	0.337	0.60 (0.26, 1.42)	0.245
Race and Ethnicity				
White	Reference		Reference	
Racial and ethnic minoritised group	0.68 (0.39, 1.20)	0.183	0.81 (0.45, 1.46)	0.483
Personal Income (CAD)				
Less than CAD 80,000	Reference		Reference	
CAD 80,000 or more	1.82 (0.82, 4.01)	0.139	2.92 (1.09, 7.77)	0.033
Depression		0.097		
Minimal (0–4)	Reference		Reference	
Mild (5–9)	0.55 (0.29, 1.03)	0.062	0.60 (0.312, 1.159)	0.128
Moderate (10–14)	1.26 (0.57, 2.79)	0.575	1.73 (0.687, 4.363)	0.245
Severe (15–27)	0.47 (0.18, 1.24)	0.127	0.74 (0.28, 1.91)	0.529

Notes. CAD = Canadian dollars, SNI = Social network index, SNI+ = Social network index accounting for online interactions.

## Data Availability

The original contributions presented in this study are included in the article/Supplementary Material. Further inquiries can be directed to the corresponding author.

## Supplementary Materials

The following supporting information can be downloaded at https://www.mdpi.com/article/10.3390/curroncol32090502/s1, Supplementary File S1: Connect 4 Health Questionnaire; Supplementary File S2: Scoring Instructions.
