# Supplementary material for: Social Networks of Adolescents and Young Adults with Cancer: A Cross-Sectional Study
Source: Curr Oncol. 2025 Sep 9;32(9):502. doi: 10.3390/curroncol32090502 (PMC12468912; doi:10.3390/curroncol32090502)
Supplement: Supplementary file 1 [file curroncol-32-00502-s001.zip › curroncol-3774591-supplementary file S1-survey.pdf]

UHN Princess Margaret Cancer Centre

CONNECT 4 HEALTH

NEEDS AND PREFERENCES FOR PEER SUPPORT AMONG ADOLESCENTS AND YOUNG ADULTS WITH CANCER

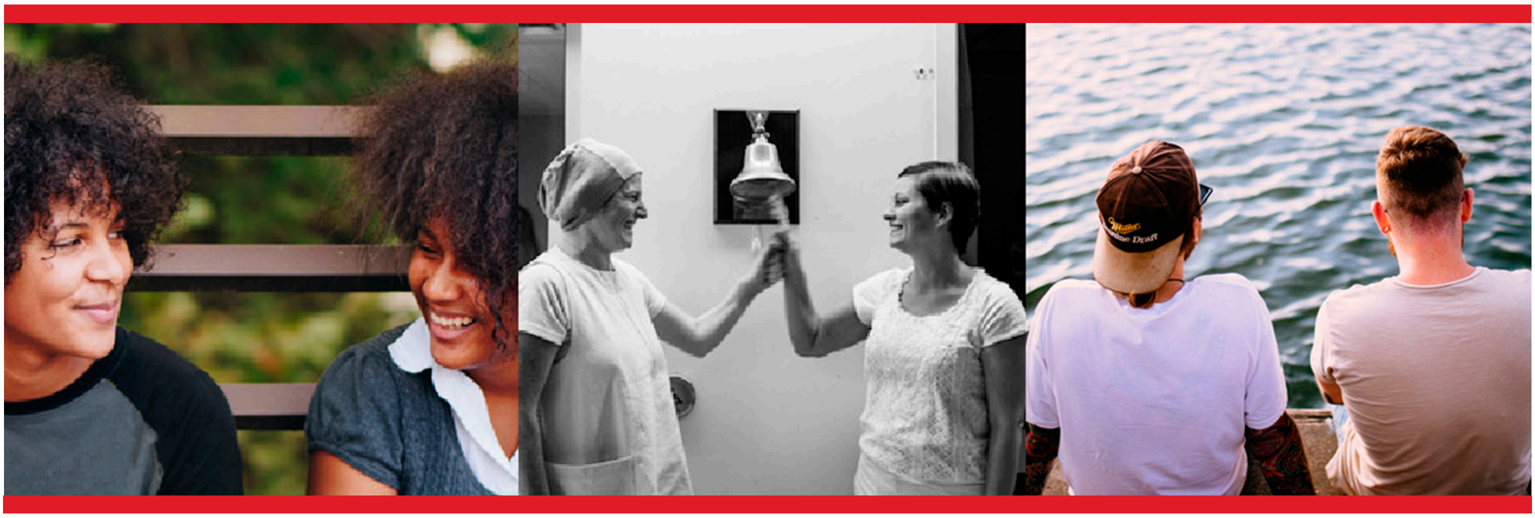

TO BE COMPLETED BY INDIVIDUALS DIAGNOSED WITH CANCER BETWEEN THE AGES OF 15-39 YEARS.

ARCC Canadian Centre for Applied Research in Cancer Control

ELLICSR Health, Wellness & Cancer Survivorship Centre

AYA Adolescent and Young Adult Program

Welcome!
Thank you for your time. This is a survey for individuals who were diagnosed with cancer as adolescents and young adults (AYA). This includes people who:

- were diagnosed with cancer between the ages of 15 and 39 years, and
- received cancer treatment within the last 10 years.

Study Title: Connect 4 Health: Needs and Requirements for a Peer Navigation Digital App for Adolescents and Young Adults (AYA) with Cancer

Principal Investigator: Jackie Bender, PhD (416) 581-8606

Co-Investigators: Norma D'Agostino, C.Psych., PhD; Abha Gupta, MD; Laura Mitchell, RN; Fuchsia Howard, RN, PhD; Sheila Garland, R.Psych., PhD; Argerie Tsimicalis, RN, PhD; Emily Drake, MA, PhD Candidate; Karine Chalifour, BSW; Anthony Marrato (Patient Partner); Nikki Leigh McKean (Patient Partner); Adriana Lombardo (Patient Partner); Priscilla Estrada (Patient Partner)

Contact Information: jackie.bender@uhnresearch.ca
*Please note that communication via e-mail is not absolutely secure. Thus, please do not communicate personal sensitive information via e-mail.

Sponsor: Canadian Centre for Applied Research in Cancer Control

Introduction:
You are being asked to take part in this research study because you were diagnosed with cancer between 15 and 39 years of age and you received cancer treatment within the last 10 years. Please read the information about the study presented in this form. The form includes details on the study's risks and benefits that you should know before you decide if you would like to take part. You should take as much time as you need to make your decision. Participation in this study is voluntary.

Background:
Peer support from people who have been through the cancer experience has been shown to be very helpful and is an important complement to the support provided by family, friends and the medical system. However, studies have shown that adolescents and young adults (AYA) with cancer may not have access to enough peer support. Many AYAs have described feeling isolated and want to be connected to peer cancer survivors. Some AYA have specifically said that they would like one-on-one support from a buddy or peer navigator to help them navigate the cancer experience.

Purpose:
The purpose of this study is to learn about AYA needs and preferences for peer support and interest in using a digital app to connect with AYA peer cancer survivors. The survey will ask about:

- Background information about you, your cancer diagnosis and any support programs that you may have accessed
- Your interest in being connected with a peer cancer survivor or navigator, and whether you are interested in becoming a peer cancer survivor or navigator
- What tools, you believe may help you connect with other peer cancer patients and survivors, like a digital app
- Your social wellbeing and overall health

By participating in this study, you can help inform cancer centres and support agencies about the peer support needs and preferences of Canadian AYAs with cancer. This information will be used to design a peer support program specifically for AYA diagnosed with cancer that may include a digital app.

Study Procedures:
You will be asked to complete one survey. The survey should take about 25 minutes to complete according to a pilot test with AYA peer cancer survivors. Up to 200 AYA cancer patients at the Princess Margaret Cancer Centre, and 200 patients recruited online though social media will be asked to complete this survey.

Risks:
We believe there are minimal risks involved in being in this study. However, sometimes when people talk about their experience they want a chance to talk to someone for support. If this happens to you, please speak to the research assistant or you can email Dr. Jackie Bender at jackie.bender@uhnresearch.ca. She will find someone for you to talk with.

Benefits:
You may or may not benefit from participating in this study. Information learned from this study will help us understand more about the important components of peer support programs and tools for AYA. Your participation in this study may also indirectly contribute to the development of a peer support digital app specifically for AYA with cancer.

Confidentiality:
Personal Information
You will be asked to provide your name and email address at the end of the survey only if you would like to:
- Enter into a draw for a gift prize
- Receive the results of the survey
- Participate in follow-up workshop to design a digital app for AYA
- Learn about becoming a peer navigator for other AYA

Your responses to this survey will be kept entirely confidential. All personal information such as your name and email address, should you choose to provide them, will be removed from the data and will be replaced with a unique identification number. A list linking the number with your name will be kept by Dr. Bender in a secure place, separate from your file. Any personal information collected about you will be kept in a secure and confidential location for 10 years.

<u>For patients completing the survey at Princess Margaret Cancer Centre:</u>
The study team will look at your personal health information from your medical record and collect only the information they need for the study, such as the details of your cancer diagnosis and treatment.

The following people may come to the hospital to look at the study records and at your personal health information to check that the information collected for the study is correct and to make sure the study is following proper laws and guidelines:
- Representatives of the University Health Network (UHN) including the UHN Research Ethics Board

All of the information gathered in this study will be used for research and education purposes only and will not be shared for any other purpose. Any identifying information will be removed from the survey. You will not be named in any reports to the sponsor, publications, or presentations that come from this study.

Voluntary Participation:
Your participation in this study is voluntary. You can stop the survey at any time and if you don't finish, we will assume that you don't want to participate in the study. In that case, we will not keep or use any of the information you have provided.

You may choose not to answer any question desired. If you want to take the survey at a later time, just click on the link again.

Withdrawal from Study:
If you decide to leave the study, you have the right to request withdrawal of information collected about you. If you would like to have your information withdrawn from the study after you complete the survey, please call Dr. Jackie Bender at (416) 581-8606.

Costs and Reimbursement:
If you complete this survey, you may provide your contact information to be entered into a draw to win one of three $100CAD VISA gift cards.

Rights as a Participant:
By completing this survey and thereby implying your consent to participate in this study, you do not give up any of your legal rights against the investigators, sponsor or involved institutions for compensation, nor does this form relieve the investigators, sponsor or involved institutions of their legal and professional responsibilities.

Conflict of Interest:
Canadian Centre for Applied Research in Cancer Control, the sponsor of this study, will reimburse the hospital and researcher for the costs of doing this study. All of these people have an interest in completing this study. Their interests should not influence your decision to participate in this study.

Questions about the study:
If you have any questions, concerns or would like to speak to the study team for any reason, please call Dr. Jackie Bender at (416) 581-8606.

If you have any questions about your rights as a research participant or have concerns about this study, call the Chair of the University Health Network Research Ethics Board (UHN REB) or the Research Ethics office number at 416-581-7849. The REB is a group of people who oversee the ethical conduct of research studies. The UHN REB is not part of the study team. Everything that you discuss will be kept confidential.

Consent:
By completing the following survey, your consent to participate in this study will be implied.

Please feel free to print this sheet for your records.

Your help means a lot to us and will be used to develop programs and services specifically for AYAs with cancer. We thank you again for taking the time to participate in this study.

Participant ID (to be completed by program staff):

Eligibility Questions

Please answer the following questions to confirm your eligibility for this study:

1. **Were you diagnosed with cancer between 15 and 39 years of age?**
   - 0 ☐ No > *Unfortunately, you are not eligible to participate in this survey.*
   - 1 ☐ Yes

2. **Have you received cancer treatment within the last 10 years?**
   - 0 ☐ No > *Unfortunately, you are not eligible to participate in this survey.*
   - 1 ☐ Yes

Part A: Cancer Specific Background Information About You

A1. Today's Date: _________/_________/__________
Day Month Year

A2. What is your age? _________years

A3. When were you first diagnosed with cancer: __________________/__________
Month Year

A4. What type of cancer were you diagnosed with?
- 1 ☐ Breast Cancer
- 2 ☐ Cervical Cancer
- 3 ☐ Colorectal Cancer
- 4 ☐ Hodgkin's Lymphoma
- 5 ☐ non-Hodgkin Lymphoma
- 6 ☐ Leukemia
- 7 ☐ Melanoma
- 8 ☐ Testicular Cancer
- 9 ☐ Thyroid Cancer
- 10 ☐ I don't know
- 11 ☐ Other. Please specify: ______________________

A5. What stage was your cancer when you were first diagnosed?
- 1 ☐ Stage 1
- 2 ☐ Stage 2
- 3 ☐ Stage 3
- 4 ☐ Stage 4
- 5 ☐ I don't know

A6. Have you had a cancer recurrence?
- 1 ☐ Yes
- 0 ☐ No

A7. Have you been diagnosed with any other types of cancer? If yes, please describe date of diagnosis and type of cancer:

Type of Cancer	Month	Year

A8. Are you living with metastatic cancer?

- 1☐ Yes
- 0☐ No

A9. Are you currently undergoing treatment?

- 1☐ Yes
- 0☐ No

A10. What types of cancer treatments have you undergone or are you currently receiving? **Check all that apply**

- 1☐ Drug or Chemotherapy
- 2☐ Hormone Therapy
- 3☐ Radiation Therapy
- 4☐ Surgery
- 5☐ Bone marrow or stem cell transplant
- 6☐ Other, please specify: ____
- 7☐ None.

A11. To the best of your knowledge, are you now cancer free?

- 1☐ Yes
- 0☐ No
- 2☐ I don't know

A7. Based on your interactions with your doctors, nurses, and other health care professionals, how would you rate the quality of care you have received since your cancer diagnosis?

1☐ Poor 2☐ Fair 3☐ Good 4☐ Very good 5☐ Excellent

Part B: Preferences for Peer Support from other Adolescents and Young Adults (AYA) Diagnosed with Cancer

B1. Since your cancer diagnosis, have you ever wanted to connect (e.g. meet in person, talk on the phone or online) with other **adolescents and young adults with cancer** **(AYA)**? This includes people who were diagnosed with cancer between the ages of 15 and 39 years.

- 1☐ Yes
- 0☐ No

B2. Since your diagnosis, has a medical professional (e.g. oncologist, nurse, psychologist) talked to you about peer support? Peer support is information, emotional or practical support from another person who has experienced cancer.

- 1☐ Yes, a medical professional initiated the conversation
- 2☐ Yes, I initiated the conversation
- 0☐ No

B3. Since your cancer diagnosis, have you ever received a referral to a peer support program (e.g. peer support online group, Young Adult Cancer Canada) from a medical professional?

- 1☐ Yes
- 0☐ No

B4. Since your diagnosis, have you connected with other AYA through any of the following sources? Select all that apply.

1☐ My health care professional
2☐ Telephone peer support call service
3☐ In-person support group
4☐ Camps, retreats, and adventure programs
5☐ Conference
6☐ Cancer organizations (e.g. Young Adult Cancer Canada -YACC, Cancer Fight Club, Stupid Cancer)
7☐ Social Media (E.g. Facebook, Twitter, Instagram, Snapchat etc.)
8☐ Online discussion forum
9☐ Digital apps that connect you with other AYA (e.g. Stupid Cancer, Upopolis)
10☐ Other, please specify: ____
11☐ I did not try to connect with other AYA

B5. Now we would like to know some of the factors that might have made it difficult for you to connect with other AYA, whether or not you tried to connect with other AYA. **Please select the BEST response.**

Factor	No problem	Somewhat of a problem	Big problem	Not sure
a. Being unsure of where or how to find other AYA	1☐	2☐	3☐	4☐
b. Being uncomfortable attending in-person support programs	1☐	2☐	3☐	4☐
c. In-person support programs were not convenient to attend	1☐	2☐	3☐	4☐
d. AYA specific in-person support programs were hard to find	1☐	2☐	3☐	4☐
e. Access to a computer or mobile device was not available	1☐	2☐	3☐	4☐
f. Using a computer or mobile device is difficult for me	1☐	2☐	3☐	4☐
g. Using social media is difficult for me	1☐	2☐	3☐	4☐
h. Discussing my health condition on social media worries me because of privacy concerns	1☐	2☐	3☐	4☐
i. Finding another AYA who I can relate to was difficult for me	1☐	2☐	3☐	4☐
j. Being concerned about hearing emotionally difficult stories	1☐	2☐	3☐	4☐
k. Being concerned about being around negative minded cancer patients	1☐	2☐	3☐	4☐
l. Being concerned about getting close to someone who might die	1☐	2☐	3☐	4☐
m. Wanting to reconnect with healthy peers and not other cancer patients	1☐	2☐	3☐	4☐
n. Other. Please specify: ______________________				
I did not try to connect with other AYA.	Yes 5☐			

B6. Now we would like to know about the **type of peer support from other AYA** that is available to you or that you may want. Check the BEST response.

	**No, not needed.**	**No, but I would like this type of peer support**	**Yes, I currently have this type of support.**	**Yes, and I would like <u>more</u> of this type of peer support**
a. Can you count on another AYA to provide you good information or advice about a problem?	1☐	2☐	3☐	4☐
b. Can you count on another AYA to provide you with emotional support (talking over problems or helping you to make a difficult decisions).	1☐	2☐	3☐	4☐
c. Can you count on another AYA to help you with practical things like daily chores, child care or getting to appointments.	1☐	2☐	3☐	4☐
d. Can you count on another AYA to hang out with and do normal social things?	1☐	2☐	3☐	4☐

B7. Overall, how satisfied are you with the peer support that you have received from other AYA so far?

1☐ Very dissatisfied
2☐ Dissatisfied
3☐ Neutral
4☐ Satisfied
5☐ Very satisfied
6☐ Not applicable. I have not received support from other AYA.

B8. Would you be interested in being connected with a **<u>peer cancer survivor</u>**? A peer cancer survivor is an AYA who has been through the cancer experience.

1☐ Yes
0☐ No. If no, what are the reasons why not?
 a☐ I don't think I need support
 b☐ I don't like to talk about my problems
 c☐ I believe I have adequate support
 d☐ Other, please specify: ______________________________

B9. Would you be interested in being connected with a **<u>peer navigator</u>**? A peer navigator is a cancer survivor who has been **<u>trained</u>** to help others through the cancer experience by providing informational, emotional and practical support.

1☐ Yes
0☐ No. If no, what are the reasons why not?
 a☐ I don't think I need support
 b☐ I don't like to talk about my problems
 c☐ I believe I have adequate support
 d☐ Other, please specify: ______________________________

B10. How important is it for you to be connected with a peer cancer survivor or navigator who has **<u>received training</u>** to support others through the cancer experience?

1☐ Not important
2☐ Slightly important
3☐ Moderately important
4☐ Important
5☐ Very important

6 ☐ Not sure

B11. How would you want to **communicate** with a peer cancer survivor or navigator? Check all that apply.

- 1☐ In-person
- 2☐ By telephone
- 3☐ By texting
- 4☐ Through email
- 5☐ On social media (Facebook, Twitter, Instagram)
- 6☐ Through video (e.g. Skype, Facetime etc.)
- 7☐ Other. Please describe:____

B12. Would you be interested in using a **digital app** to connect and communicate with a peer cancer survivor or navigator? You could view the profiles of AYA that match your selection criteria (e.g. age, cancer diagnosis) and choose the AYA who you would like to talk to.

- 1☐ Yes
- 0☐ No. If no, what are the reasons why not?________________________________

B13. In this digital app would you want to be able to communicate. **Check all that apply:**

- 1☐ One-on-one with one peer cancer survivor or navigator
- 2☐ One-on-one with one or more peer cancer survivors or navigators
- 3☐ In a group with many other AYA, like an online discussion forum
- 4☐ Other. Please describe:____

B14. How important would it be for you to be **matched** with a peer cancer survivor or navigator who is similar to you in terms of the following characteristics? **Check the BEST response for each row.**

	Not important at all	Somewhat important	Very Important	Not sure
a. Age at diagnosis	1☐	2☐	3☐	4☐
b. Current age	1☐	2☐	3☐	4☐
c. Type of cancer	1☐	2☐	3☐	4☐
d. Stage of disease (e.g. 1, 2, 3, 4)	1☐	2☐	3☐	4☐
e. Treatments received/considering	1☐	2☐	3☐	4☐
f. Specific concerns (e.g. side effects, return to school/work)	1☐	2☐	3☐	4☐
g. Hospital where you were treated	1☐	2☐	3☐	4☐
h. Geographic region where you live	1☐	2☐	3☐	4☐
i. Gender	1☐	2☐	3☐	4☐
j. Sexual orientation	1☐	2☐	3☐	4☐
k. Race/Ethnicity	1☐	2☐	3☐	4☐
l. Relationship Status (e.g. single, married)	1☐	2☐	3☐	4☐
m. Education	1☐	2☐	3☐	4☐
n. Personality style	1☐	2☐	3☐	4☐
o. Coping style	1☐	2☐	3☐	4☐
p. Hobbies/interests	1☐	2☐	3☐	4☐
q. Religion/spirituality	1☐	2☐	3☐	4☐
r. Other. Please specify:________________________________				

B15. **When** would it be most helpful to be connected with a peer cancer survivor or navigator? Check all that apply.

1☐ During diagnosis
2☐ Before treatment
3☐ During treatment
4☐ After treatment
5☐ If cancer recurs or spreads
6☐ Other. Please specify:______________________________

B16. Now, we would like to know what type of support you would like to receive from a peer cancer survivor or navigator. Please rate the importance of each of the following types of support from being very important to not important at all.

	**Not important at all**	**Slightly important**	**Moderately important**	**Important**	**Very important**
a. **Informational support** (e.g. Information about cancer, its treatment, side effects, AYA-specific resources, hospital programming, transition to survivorship)	1☐	2☐	3☐	4☐	5☐
b. **Emotional support** (e.g. Discussion of experiences, validation of feelings and concerns, and coping with the cancer and its treatment and its impact on your life, your relationships and your plans etc.)	1☐	2☐	3☐	4☐	5☐
c. **Practical support** (e.g. Assistance to attend appointments, arrange leave/return to school or work, financial support, childcare, travelling etc.)	1☐	2☐	3☐	4☐	5☐
d. **Social companionship** (e.g. Someone to hang out with and do normal social things)	1☐	2☐	3☐	4☐	5☐
e. Other. Please specify:______________________________					

B17. Would you be interested in volunteering to be a peer navigator to support other AYA through the cancer journey? (You would be provided training and professional support to be a peer navigator. You could communicate with your matches online, by phone or in-person.)

The primary role of a Peer Navigator is to:

- Assist patients to identify needs and overcome barriers to getting their needs met
- Empower patients by working with them to identify their own strengths, abilities and coping strategies
- Discuss experiences, validate feelings and concerns

1☐ Yes
0☐ No. If no, what are the reasons why not:______________________________

B18. Have you ever provided peer support to other people affected by cancer?

1☐ Yes
0☐ No

B19. Have you received training to provide peer support to other people affected by cancer?

[1] ☐ Yes. If yes, please describe:____

[0] ☐ No

B20. Would you be willing to attend a peer navigator training course to become a peer navigator?

This course would provide you with the knowledge, skills and competencies to be a peer navigator for other AYA. It would be delivered online over the course of 6 weeks, with 1 to 2 in-person workshops. It would require a commitment of about 4 hours per week, or 24 hours in total. You would be provided a certificate at the completion of the course.

[1] ☐ Yes

[0] ☐ No. If No, please explain why not:____

Part C: Overall Health and Wellbeing

C1. The next questions ask about your health. By health we mean not only the absence of disease or injury, but also physical, mental and social well-being. In general, how would you rate your overall health?

[1] ☐ Poor [2] ☐ Fair [3] ☐ Good [4] ☐ Very good [5] ☐ Excellent

C2. Using a scale of 0-10, where 0 means "Very dissatisfied" and 10 means "Very Satisfied", how do you feel about your life as a whole right now?

Very Dissatisfied — Very Satisfied

0	1	2	3	4	5	6	7	8	9	10

C3. Please indicate what kind of overall impact your cancer has had on each of the following areas of your life. If a question doesn't apply to you, select "Does not apply".

Overall impact of cancer on your…	Very negative impact	Somewhat negative impact	No impact	Somewhat positive impact	Very positive impact	Does not apply
a. Relationship with your mother	1 ☐	2 ☐	3 ☐	4 ☐	5 ☐	10 ☐
b. Relationship with your father	1 ☐	2 ☐	3 ☐	4 ☐	5 ☐	10 ☐
c. Relationship with your brothers or sisters	1 ☐	2 ☐	3 ☐	4 ☐	5 ☐	10 ☐
d. Relationship with your spouse, partner, boyfriend or girlfriend	1 ☐	2 ☐	3 ☐	4 ☐	5 ☐	10 ☐
e. Relationship with your child/children	1 ☐	2 ☐	3 ☐	4 ☐	5 ☐	10 ☐
f. Relationship with friends	1 ☐	2 ☐	3 ☐	4 ☐	5 ☐	10 ☐
g. Dating	1 ☐	2 ☐	3 ☐	4 ☐	5 ☐	10 ☐
h. Plans for getting married	1 ☐	2 ☐	3 ☐	4 ☐	5 ☐	10 ☐

Overall impact of cancer on your…	Very negative impact	Somewhat negative impact	No impact	Somewhat positive impact	Very positive impact	Does not apply
i. Sexual function/intimate relations	1☐	2☐	3☐	4☐	5☐	10☐
j. Plans for having children	1☐	2☐	3☐	4☐	5☐	10☐
k. Spirituality and religious beliefs	1☐	2☐	3☐	4☐	5☐	10☐
l. Plans for the future and goal setting	1☐	2☐	3☐	4☐	5☐	10☐
m. Feelings about the appearance of your body	1☐	2☐	3☐	4☐	5☐	10☐
n. Confidence in your ability to take care of your health	1☐	2☐	3☐	4☐	5☐	10☐
o. Control over your life	1☐	2☐	3☐	4☐	5☐	10☐
p. Plans for education	1☐	2☐	3☐	4☐	5☐	10☐
q. Plans for work	1☐	2☐	3☐	4☐	5☐	10☐
r. Financial situation	1☐	2☐	3☐	4☐	5☐	10☐

C4. During the past 4 weeks, have you experienced any of the following problems, whether related to your cancer or not?

		No	Yes
a.	Nausea or vomiting	0☐	1☐
b.	Frequent or severe stomach pain	0☐	1☐
c.	Diarrhea or constipation	0☐	1☐
d.	Pain in your joints (for example, knees, ankles, elbows) or bones	0☐	1☐
e.	Weight loss	0☐	1☐
f.	Weight gain	0☐	1☐
g.	Frequent or severe fevers	0☐	1☐
h.	Hot flashes	0☐	1☐
i.	Tingling, weakness, or clumsiness of the hands or feet	0☐	1☐
j.	Frequent or severe headaches	0☐	1☐
k.	Frequent or severe mouth sores that impact your eating and drinking	0☐	1☐
l.	Problems with memory, attention, or concentration	0☐	1☐

C5. During the past 2 weeks, how often have you been bothered by any of the following problems?

	Not at all	Several days	More than half the days	Nearly everyday
1. Feeling nervous, anxious or on edge	0☐	1☐	2☐	3☐
2. Not being able to stop or control worrying	0☐	1☐	2☐	3☐
3. Worrying to much about different things	0☐	1☐	2☐	3☐
**4.** Trouble relaxing	0☐	1☐	2☐	3☐
5. Being so restless that it is hard to sit still	0☐	1☐	2☐	3☐
6. Becoming easily annoyed or irritable	0☐	1☐	2☐	3☐
7. Feeling afraid as if something awful might happen	0☐	1☐	2☐	3☐

C6. During the past 2 weeks, how often have you been bothered by any of the following problems?

	Not at all	Several days	More than half the days	Nearly everyday
1. Little interest or pleasure in doing things	0☐	1☐	2☐	3☐
2. Feeling down, depressed or hopeless	0☐	1☐	2☐	3☐
3. Trouble falling or staying asleep, or sleeping too much	0☐	1☐	2☐	3☐
**4.** Feeling tired or having little energy	0☐	1☐	2☐	3☐
5. Poor appetite or overeating	0☐	1☐	2☐	3☐
6. Feeling bad about yourself – or that you are a failure or have let yourself or your family down	0☐	1☐	2☐	3☐
7. Trouble concentrating on things, such as reading the newspaper or watching television	0☐	1☐	2☐	3☐
8. Moving or speaking so slowly that other people could have noticed? Or the opposite – being so fidget or restless that you gave been moving around a lot more than usual	0☐	1☐	2☐	3☐
9. Thoughts that you be better off dead or of hurting yourself in some way	0☐	1☐	2☐	3☐

If you checked off any of these problems, how difficult have these problems made if for you to do your work, take care of things at home, or get along with other people?

1☐ Not at all
2☐ Somewhat difficult
3☐ Very difficult
4☐ Extremely difficult

C7. The next questions are about **how you feel** about different aspects of your life. Please select the BEST response.

	I often feel this way	I sometimes feel this way	I rarely feel this way	I never feel this way
1. I am unhappy doing so many things alone.	1☐	2☐	3☐	4☐
2. I have nobody to talk to	1☐	2☐	3☐	4☐
3. I cannot tolerate being so alone	1☐	2☐	3☐	4☐
4. I lack companionship	1☐	2☐	3☐	4☐
5. I feel as if nobody really understands me	1☐	2☐	3☐	4☐
6. I find myself waiting for people to call or write	1☐	2☐	3☐	4☐
7. There is no one I can turn to	1☐	2☐	3☐	4☐
8. I am not longer close to anyone	1☐	2☐	3☐	4☐
9. My interests and ideas are not shared by those around me	1☐	2☐	3☐	4☐
10. I feel left out	1☐	2☐	3☐	4☐
11. I feel completely alone	1☐	2☐	3☐	4☐
12. I am unable to reach out and communicate with those around me	1☐	2☐	3☐	4☐
13. My social relationships are superficial	1☐	2☐	3☐	4☐
14. I feel starved for company	1☐	2☐	3☐	4☐
15. No one really knows me well	1☐	2☐	3☐	4☐
16. I feel isolated from others	1☐	2☐	3☐	4☐
17. I am unhappy being so withdrawn	1☐	2☐	3☐	4☐
18. It is difficult for me to make friends	1☐	2☐	3☐	4☐
19. I feel shut out and excluded by others	1☐	2☐	3☐	4☐
20. People are around me but not with me	1☐	2☐	3☐	4☐

C8. The next questions are about your **current relationships** with friends, family members, co-workers, community members etc. Please indicate to what extent each statement describes your current relationships with other people.

	Strongly agree	Agree	Disagree	Strongly Disagree
1. There are people I can depend on to help	0☐	1☐	2☐	3☐
2. There are people who enjoy the same activities as I do.	0☐	1☐	2☐	3☐
3. I have close relationships that provide me with a sense of emotional security and wellbeing.	0☐	1☐	2☐	3☐
4. There is someone I could talk to about important decisions in my life.	0☐	1☐	2☐	3☐

	Strongly agree	Agree	Disagree	Strongly Disagree
5. I have relationships where my competence and skill are recognized.	1☐	2☐	3☐	4☐
6. There is a trustworthy person I could turn to for advice if I were having problems.	1☐	2☐	3☐	4☐
7. I feel part of a group of people who share my attitudes and beliefs.	1☐	2☐	3☐	4☐
8. I feel a strong emotional bond with at least one other person.	1☐	2☐	3☐	4☐
9. There are people who admire my talents and abilities.	1☐	2☐	3☐	4☐
10. There are people I can count on in an emergency.	1☐	2☐	3☐	4☐

C9. The following questions ask about your contact with your **<u>social network</u>**. Please read the following questions and select the response that most closely describes your <u>current</u> situation.

a. What is your relationship status?

1☐ Single
2☐ Married or living with a life partner
3☐ In a relationship, but not living with a life partner

b. How many *close friends* do you have? (people that you feel at ease with, can talk to about private matters and can call on for help.)

0☐ None
1☐ 1 or 2
2☐ 3 to 5
3☐ 6 to 9
4☐ 10 or more

c. How many of these *close friends* do you see at least once a month?

0☐ None
1☐ 1 or 2
2☐ 3 to 5
3☐ 6 to 9
4☐ 10 or more

d. How many of these *close friends* do you talk to <u>by phone</u> at least once a month?

0☐ None
1☐ 1 or 2
2☐ 3 to 5
3☐ 6 to 9
4☐ 10 or more

e. How many of these *close friends* do you communicate with by <u>text, email, or other ways online</u> at least once a month?

0☐ None
1☐ 1 or 2
2☐ 3 to 5
3☐ 6 to 9
4☐ 10 or more

f. How many *relatives* do you have that you feel close to?

0☐ None
1☐ 1 or 2
2☐ 3 to 5
3☐ 6 to 9
4☐ 10 or more

g. How many of these *relatives* do you see at least once a month?

0☐ None
1☐ 1 or 2
2☐ 3 to 5
3☐ 6 to 9
4☐ 10 or more

h. How many of these *relatives* do you talk to <u>by phone</u> at least once a month?

0☐ None
1☐ 1 or 2
2☐ 3 to 5
3☐ 6 to 9
4☐ 10 or more

i. How many of these *relatives* do you communicate with by <u>text, email, or other ways online</u> at least once a month?

0☐ None
1☐ 1 or 2
2☐ 3 to 5
3☐ 6 to 9
4☐ 10 or more

j. Do you belong to any of these kinds of social groups or organizations?

1☐ Professional association or business group
2☐ Organized sports team
3☐ Neighborhood or block association
4☐ A service organization (like a Rotary, Red Cross or food bank)
5☐ A religious organization like a church, synagogue, mosque.
6☐ A cultural organization (like a choir, amateur theatre or comedy group)
7☐ Another kind of social group or organization. Please describe:___________________________________
8☐ None.

k. About how often do you go to religious or spiritual meetings or services?

0☐ Never
1☐ Once or twice a year
2☐ Every few months
3☐ Once or twice a month
4☐ Once a week
5☐ More than once a week

I. Now, we would like to know whether your contact with family, friends or participation in social groups has changed since your diagnosis with cancer. Please select the BEST response.

	**More often**	**The same amount**	**Less often**	**Does Not Apply**
1. Since my cancer diagnosis, I see or	0 ☐	1 ☐	2 ☐	3 ☐
2. Since my cancer diagnosis, I see or communicate with my **close relatives…**	0 ☐	1 ☐	2 ☐	3 ☐
3. Since my cancer diagnosis, I participate in	0 ☐	1 ☐	2 ☐	3 ☐
**4.** Since my cancer diagnosis, I participate in **religious or spiritual meetings or services…**	0 ☐	1 ☐	2 ☐	3 ☐

C10. We are interested in how confident you are that you can do certain things. If you select a “7”, you are totally confident that you can do that behaviour. If you select a “1” you are not at all confident that you can do that behavior. Numbers in the middle mean that you are somewhat confident that you can do that behavior. Be sure your ratings are about your <u>confidence even if you have not done the behavior in the past</u>.

	**Not at all confident**		**Moderately Confident**				**Totally confident**
	**1**	**2**	**3**	**4**	**5**	**6**	**7**
1. Maintaining independence	1 ☐	2 ☐	3 ☐	4 ☐	5 ☐	6 ☐	7 ☐
2. Maintaining a positive attitude	1 ☐	2 ☐	3 ☐	4 ☐	5 ☐	6 ☐	7 ☐
3. Accepting that I have cancer	1 ☐	2 ☐	3 ☐	4 ☐	5 ☐	6 ☐	7 ☐
4. Maintaining activities (school, work, home, social)	1 ☐	2 ☐	3 ☐	4 ☐	5 ☐	6 ☐	7 ☐
5. Asking nurses questions	1 ☐	2 ☐	3 ☐	4 ☐	5 ☐	6 ☐	7 ☐
6. Trying to be calm throughout treatments and not allowing scary thoughts to upset me	1 ☐	2 ☐	3 ☐	4 ☐	5 ☐	6 ☐	7 ☐
7. Seeking support from people & groups outside the family	1 ☐	2 ☐	3 ☐	4 ☐	5 ☐	6 ☐	7 ☐
8. Maintaining a daily routine	1 ☐	2 ☐	3 ☐	4 ☐	5 ☐	6 ☐	7 ☐
9. Asking my health care professionals questions	1 ☐	2 ☐	3 ☐	4 ☐	5 ☐	6 ☐	7 ☐
10. Using spiritual/religious beliefs as a source of coping	1 ☐	2 ☐	3 ☐	4 ☐	5 ☐	6 ☐	7 ☐
11. Putting things out of my mind at times	1 ☐	2 ☐	3 ☐	4 ☐	5 ☐	6 ☐	7 ☐
12. Trying to be calm while receiving treatment (chemotherapy, radiation)	1 ☐	2 ☐	3 ☐	4 ☐	5 ☐	6 ☐	7 ☐

	Not at all confident	Moderately Confident					Totally confident
	1	2	3	4	5	6	7
13. Coping with physical changes	1☐	2☐	3☐	4☐	5☐	6☐	7☐
14. Learning to "let things go" at times	1☐	2☐	3☐	4☐	5☐	6☐	7☐
15. Actively participating in treatment decisions	1☐	2☐	3☐	4☐	5☐	6☐	7☐
16. Using spirituality/religion to give my life meaning	1☐	2☐	3☐	4☐	5☐	6☐	7☐
17. Sharing my worries or concerns with others	1☐	2☐	3☐	4☐	5☐	6☐	7☐
18. Maintaining hope using spirituality/religion	1☐	2☐	3☐	4☐	5☐	6☐	7☐
19. Keeping busy with activities	1☐	2☐	3☐	4☐	5☐	6☐	7☐
20. Maintaining a sense of humour	1☐	2☐	3☐	4☐	5☐	6☐	7☐
21. Accepting physical changes or limitations caused by cancer treatment	1☐	2☐	3☐	4☐	5☐	6☐	7☐
22. Seeking social support	1☐	2☐	3☐	4☐	5☐	6☐	7☐
23. Maintaining hope	1☐	2☐	3☐	4☐	5☐	6☐	7☐
24. Using spiritual/religious beliefs to understand my reasons for living/ survival	1☐	2☐	3☐	4☐	5☐	6☐	7☐
25. Asking physicians questions	1☐	2☐	3☐	4☐	5☐	6☐	7☐
26. Coping with aches and pains	1☐	2☐	3☐	4☐	5☐	6☐	7☐
27. Managing nausea and vomiting (whether or not I have these problems in the past)	1☐	2☐	3☐	4☐	5☐	6☐	7☐

C11a. Since your cancer diagnosis, you have used or needed any of the following **informational resources**.

	Have used and would like to use more.	Have used and have no further need.	Have NOT used but would like to.	Have NOT used and have no need.
a. Information about illness, treatment, risks for recurrence or second cancers	1☐	2☐	3☐	4☐
b. Internet sites that offer cancer education or support for AYA	1☐	2☐	3☐	4☐
c. Information about infertility or options for having children	1☐	2☐	3☐	4☐
d. Information or counseling about exercise and physical fitness	1☐	2☐	3☐	4☐
e. Information or counseling about diet and nutrition	1☐	2☐	3☐	4☐
f. Information or counseling about return to school or work	1☐	2☐	3☐	4☐

C11b. Since your cancer diagnosis, have used or needed any of the following **emotional support services.**

	Have used and would like to use more.	Have used and have no further need.	Have NOT used but would like to.	ave NOT used and have no need.
g. Community centres, camps, retreats, or adventure programs that offer cancer education or support for AYA	1☐	2☐	3☐	4☐
h. Counseling by mental health professionals (such as psychiatrists, social workers and psychologists) to help with anxiety, depression or emotional stress	1☐	2☐	3☐	4☐
i. Counseling or guidance related to sexuality or intimacy	1☐	2☐	3☐	4☐
j. Religious or spiritual support or counseling	1☐	2☐	3☐	4☐
k. Family counseling focusing on family relationships	1☐	2☐	3☐	4☐

C11c. Since your cancer diagnosis, have used or needed any of the following **practical support services.**

	Have used and would like to use more.	Have used and have no further need.	Have NOT used but ould like to.	Have NOT used and have no need.
l. Help with understanding financial support, health insurance, disability or social security	1☐	2☐	3☐	4☐
m. Child care	1☐	2☐	3☐	4☐
n. Infertility treatment (including sperm banking, egg harvesting, artificial insemination, in vitro fertilization, and surrogacy)	1☐	2☐	3☐	4☐
o. Transportation assistance (such as taxi vouchers, etc.)	1☐	2☐	3☐	4☐
p. Complementary and alternative health care (herbal treatment, acupuncture, meditation etc).	1☐	2☐	3☐	4☐

Part D: Background Information About You

In this final section, we are interested in some background information about you. We want to ensure that people with a range of backgrounds are involved in this study.

D1. What is the highest level of education that you have completed?

1☐ Elementary school
2☐ Some high school
3☐ Completed high school
4☐ Some college or vocational school
5☐ Completed college or vocational school
6☐ Some university
7☐ Completed university
8☐ Other. Please specify:______________________________

D2. What is your current school or employment status? Please select all that apply.

1 ☐ Part-time student
2 ☐ Full-time student
3 ☐ Working part-time
4 ☐ Working full-time
5 ☐ Unemployed and looking for work
6 ☐ Unemployed and not looking for work
7 ☐ Full-time homemaker or family caregiver
8 ☐ Other. Please specify:____________________

D3. Has your school/employment status changed because of your cancer or its treatment? Please select all that apply.

1 ☐ It has not changed because of my cancer or its treatment
2 ☐ I was unable to work and went on disability benefits.
3 ☐ I quit working completely
4 ☐ I quit going to school completely
5 ☐ I changed my work status from full-time to part-time
6 ☐ I changed my school status from full-time to part-time
7 ☐ I took more than 2 weeks total time off from work
8 ☐ I took more than 2 weeks total time off from school
9 ☐ I asked for work flexibility (e.g. worked remotely, changed my hours)
10 ☐ I closed my business
11 ☐ Other. Please specify: ________________________

D4. Do you currently live alone or with others?

1 ☐ Live alone
2 ☐ Live with others (e.g. parent, roommate, spouse, partner, brother, sister, children)

D5. Are you now responsible for raising any children under the age of 18?

1 ☐ Yes
0 ☐ No

D6. What is your sex?

1 ☐ Male
2 ☐ Female
3 ☐ Prefer not to answer

D7. What is your gender?

1 ☐ Male
2 ☐ Female
3 ☐ TransMale/Transman
4 ☐ TransFemale/Transwoman
5 ☐ Genderqueer
6 ☐ Other. Please specify:_________________________
7 ☐ Prefer not to answer

D8. How would you describe your sexuality?

1 ☐ Heterosexual
2 ☐ Homosexual
3 ☐ Bisexual
4 ☐ Prefer not to answer
5 ☐ Other (Please specify:_________________________

D9. Were you born in Canada?

1 ☐ Yes
2 ☐ No. If no, in which country were you born?_____________________

D10. People in Canada come from many different backgrounds. Are you: (check all that apply)

1 ☐ Aboriginal (e.g. First Nations, Metis, Inuit)
2 ☐ White
3 ☐ South Asian (e.g. Indian, Pakistani, Sri Lankan…)
4 ☐ Chinese
5 ☐ Black
6 ☐ Filipino
7 ☐ Latin American
8 ☐ Arab
9 ☐ Southeast Asian (e.g. Vietnamese, Cambodian, Malaysian, Laotian,)
10 ☐ West Asian (e.g. Iranian, Afghan)
11 ☐ Korean
12 ☐ Japanese
13 ☐ African
14 ☐ Caribbean
15 ☐ Other. Please specify:_____________________

D11. What language do you speak most often at home?

1 ☐ English
2 ☐ French
3 ☐ Other. Please specify: _______________________

D12. In which province or territory do you live?

1 ☐ Newfoundland and Labrador
2 ☐ Prince Edward Island
3 ☐ Nova Scotia
4 ☐ New Brunswick
5 ☐ Quebec
6 ☐ Ontario
7 ☐ Manitoba
8 ☐ Saskatchewan
9 ☐ Alberta
10 ☐ British Columbia
11 ☐ Yukon
12 ☐ Northwest Terrioties
13 ☐ Nunavut
14 ☐ I do not live in Canada

D13. In what type of setting do you live most of the year?

1 ☐ Urban or Suburban (city)
2 ☐ Town or Rural (country)

D14. Which of the following categories best describes your personal income in 2017?

1 ☐ Less than $20,000
2 ☐ $20,000 to less than $40,000
3 ☐ $40,000 to less than $60,000
4 ☐ $60,000 to less than $80,000
5 ☐ $80,000 or more
6 ☐ No income. (I am not working)
7 ☐ Prefer not to answer

Thank you for completing this survey.
If you are interested in participating in a prize draw, receiving the results, learning about becoming a peer navigator, or participating in a workshop to help us design a digital app please turn the page!

For the following, questions, please indicate if you are interested by checking the appropriate box. If you answer "Yes" to any of these questions, please provide your contact information (first and last name and email address) in the space provided below.

**Follow-up Question 1:** Would you like to be entered in a draw to win one of three $100CDN VISA gift cards?	☐ Yes	☐ No
**Follow-up Question 2:** Would you like to receive email notifications of any reports resulting from this survey?	☐ Yes	☐ No
**Follow-up Question 3:** Are you interested in participating in a design workshop to help us build a digital app to connect AYA cancer patients with AYA peer cancer survivors? This would involve participating in three 2 to 3 hour focus group workshops with other AYA cancer patients and survivors. These workshops would be held at the Toronto General Hospital in downtown Toronto. You would receive up to $50 for your participation and reimbursement for your travel. Check this box if you would like to participate or are interested in learning more.	☐ Yes	☐ No
**Follow-up Question 4**: Are you interested in learning more about becoming a peer navigator?	☐ Yes	☐ No

Name:______________________________Email Address:_____________________________

*Please note that the security of email messages is not guaranteed. Messages sent to, or from, your care provider may be seen by others using the Internet. Email is easy to forge, easy to forward, and may exist indefinitely. For this reason, it is recommended that you do not use email to discuss information you think is sensitive.

Participant ID (to be completed by program staff):
