# Supplementary material for: Social Networks of Adolescents and Young Adults with Cancer: A Cross-Sectional Study
Source: Curr Oncol. 2025 Sep 9;32(9):502. doi: 10.3390/curroncol32090502 (PMC12468912; doi:10.3390/curroncol32090502)
Supplement: Supplementary file 1 [file curroncol-32-00502-s001.zip › curroncol-3774591-supplementary file S2-SNI scoring instructions.pdf]

S2. Scoring Instructions

Domain 1. Marital Status

- Single = 0 and all other responses = 1 [1,2].

Domain 2. Close Contacts

The response options for each question were 0, 1-2, 3-5, 6-9, and 10+. Using the average number of each response option, the sum of in-person and over the telephone close contacts per month was computed.

1. Frequency of close contacts in-person or over the phone
    - Less than 13 close contacts per month = 0 and 13 or more close contacts per month = 1 [1,3].
2. Frequency of close contacts in-person, over the phone, and online
    - Less than 13 close contacts per month = 0 and 13 or more close contacts per month = 1 [1,3].

Domain 3. Group Participation

- None = 0 and all other responses = 1 [1,2].

Domain 4. Religious Participation

- Attendance less than or equal to "every few months" = 0 and attendance equal to or greater than "once or twice a month" = 1 [2,4].

Outcome 1. Social Network Index

- Sum the scores of Marital Status, Close Contacts 1 (accounting for the number of close contacts in-person and/or over the telephone per month), Group Participation, and Religious Participation.

Outcome 2. Social Network Index Including Online Interactions

- Sum the scores of Marital Status, Close Contacts 2 (accounting for the number of close contacts in-person, over the telephone and/or online per month), Group Participation, and Religious Participation.

References

1. Ford, E.S.; Loucks, E.B.; Berkman, L.F. Social Integration and Concentrations of C-Reactive Protein among US Adults. *Ann. Epidemiol.* **2006**, *16*, 78–84, doi:10.1016/j.annepidem.2005.08.005.
2. Loucks, E.B.; Sullivan, L.M.; D'Agostino, R.B.; Larson, M.G.; Berkman, L.F.; Benjamin, E.J. Social Networks and Inflammatory Markers in the Framingham Heart Study. *J. Biosoc. Sci.* **2006**, *38*, 835–842, doi:10.1017/S0021932005001203.
3. Pantell, M.; Rehkopf, D.; Jutte, D.; Syme, S.L.; Balmes, J.; Adler, N. Social Isolation: A Predictor of Mortality Comparable to Traditional Clinical Risk Factors. *Am. J. Public Health* **2013**, *103*, 2056–2062, doi:10.2105/AJPH.2013.301261.
4. Zhong, R.; Zhang, H.; Chen, Q.; Guo, X.; Han, Y.; Lin, W. Social Isolation and Associated Factors in Chinese Adults With Epilepsy: A Cross-Sectional Study. *Front. Neurol.* **2021**, *12*, 813698, doi:10.3389/fneur.2021.813698.
